# Antioxidant and Anti-Inflammatory Activities of Cutlassfish Head Peptone in RAW 264.7 Macrophages

**DOI:** 10.3390/antiox14030286

**Published:** 2025-02-27

**Authors:** Su-Jin Lee, Jeonghyeon Im, Svini Dileepa Marasinghe, Eunyoung Jo, Minthari Sakethanika Bandara, Youngdeuk Lee, Jaewon Lee, Gun-Hoo Park, Chulhong Oh

**Affiliations:** 1 Jeju Bio Research Center, Korea Institute of Ocean Science and Technology (KIOST), Jeju 63349, Republic of Korea; sujinlee5699@gmail.com (S.-J.L.); imjh0125@kiost.ac.kr (J.I.); svinidileepa@gmail.com (S.D.M.); jey8574@kiost.ac.kr (E.J.); minthari@kiost.ac.kr (M.S.B.); lyd1981@kiost.ac.kr (Y.L.); rjwonlee89@kiost.ac.kr (J.L.); 2 University of Science and Technology, Gajeong-ro, Yuseong-gu, Daejeon 34113, Republic of Korea

**Keywords:** cutlassfish head peptone (CP), fishery by-products, antioxidant activity, anti-inflammatory, MAPK pathway

## Abstract

The rapid growth of the fisheries industry has resulted in numerous by-products, usually called waste, causing environmental and economic challenges. Recent advances in valorization techniques have highlighted the potential of these by-products as sources of bioactive compounds. This study aimed to investigate the antioxidant and anti-inflammatory activities of cutlassfish *(Trichiurus lepturus)* head peptone (CP) in lipopolysaccharide (LPS)-stimulated RAW 264.7 macrophages. CP exhibited significant antioxidant activity, reducing ABTS and DPPH radical scavenging activity by up to 79.66% and 64.69%, respectively, with a maximum ferric-reducing antioxidant power (FRAP) value of 224.54 μM. CP enhanced macrophage proliferation (33.3%) and significantly mitigated LPS-induced oxidative and inflammatory responses, reducing nitric oxide (NO) production (60%) and reactive oxygen species levels (49.14%). CP suppressed the expression of inflammatory mediators, including inducible nitric oxide synthase (iNOS) and cyclooxygen-ase-2, and selectively inhibited the pro-inflammatory cytokines interleukin (IL)-1β and IL-6. Western blot analysis revealed that CP inhibited the phosphorylation of mitogen-activated protein kinases, including ERK, JNK, and p38, highlighting its role in modulating upstream inflammatory signaling pathways. CP exhibited significant antioxidant effects, particularly in scavenging ABTS and DPPH radicals, as well as reducing oxidative stress markers and inflammatory responses in LPS-stimulated macrophages. These findings suggest its potential not only as a therapeutic agent for conditions related to chronic inflammation, such as cardiovascular diseases and arthritis, but also as a functional ingredient in foods and nutraceuticals aimed at alleviating inflammation-related disorders.

## 1. Introduction

The rapid development of the fisheries industry has significantly increased the abundance of fishery by-products. During the production, processing, and distribution of fish, shellfish, and crustaceans, by-products such as fish heads, entrails, bones, and skins are generated, constituting 40–70% of the total fish weight [1]. The annual amount of discarded fishery by-products is estimated to be approximately 1.09 million tons, including fish heads, entrails, bones, and skins, contributing to environmental and economic challenges [2]. These by-products are often disposed of through landfilling or incineration, leading to environmental issues such as greenhouse gas emissions, water pollution, and habitat degradation [3]. Moreover, the inefficient utilization of these materials results in substantial economic losses [3]. However, various recent recycling and valorization methods have been explored to transform these materials into valuable resources [4].

This shift is largely driven by the abundant bioactive compounds in fishery by-products, such as collagen, polyunsaturated fatty acids, chitosan, and astaxanthin [5,6,7]. These compounds have garnered significant attention from academia and industry due to their economic and physiological benefits [8,9]. Notably, the protein content in fish waste is comparable to that of edible parts [10]. Studies have reported that fish heads, skins, and frames contain 15–25% protein, which is comparable to the 18–22% protein content typically found in edible parts, such as fillets [11,12]. This high protein content highlights the potential of fishery by-products as a valuable source for bioactive compound extraction, contributing to sustainability within the fisheries industry. Among them, fish protein hydrolysates are produced through enzymatic hydrolysis, which has emerged as a highly efficient utilization method for these proteins [13].

Fish protein hydrolysates comprise small peptides and free amino acids and exhibit various biological activities, including antioxidant, antimicrobial, antihypertensive, anti-obesity, and anti-inflammatory properties [4,14]. Consequently, fish protein hydrolysates are applicable in diverse fields such as food (protein supplementation), animal feed (for pets and livestock), cosmetics (skincare products), pharmaceuticals and biotechnology (drug delivery and health supplements), and agriculture (organic fertilizers) [15].

Chronic inflammation plays a critical role in the pathogenesis of various diseases, including cardiovascular diseases, diabetes, and autoimmune disorders [16]. Hence, inflammation control is garnering significant attention, and bioactive compounds that reduce inflammation through dietary or therapeutic interventions are focused on. Recent studies have demonstrated the potential of peptones extracted from fish by-products in modulating inflammatory responses by inhibiting pro-inflammatory cytokines and blocking crucial inflammatory pathways [17,18]. These peptones effectively reduce the expression of inflammatory mediators such as tumor necrosis factor (TNF)-α, interleukin (IL)-6, and cyclooxygenase (COX)-2, alleviating inflammation-related symptoms [17,18].

Cutlassfish *(Trichiurus lepturus)* is a commercially significant demersal marine species in temperate and tropical waters and is commonly consumed in East Asia [19,20]. However, cutlassfish heads have limited culinary applications and are largely discarded as waste. Cutlassfish is rich in proteins, polyunsaturated fatty acids, and other bioactive compounds, making it an ideal candidate for bioactive peptide extraction [21]. Previous studies have demonstrated that marine-derived peptides from fish such as tuna and salmon provide significant health benefits, ranging from enhanced immune function to improved cardiovascular health [22,23]. Moreover, bioactive peptides from marine sources have been linked to protective effects against neurodegenerative diseases, metabolic disorders, and immune modulation, reinforcing the significance of cutlassfish-derived hydrolysates [24]. Recent research has also reported that cutlassfish by-products exhibit antihypertensive, fatigue-reducing, and antioxidant properties, further emphasizing their functional potential and broad physiological benefits [25,26]. In our previous study, we demonstrated that enzymatic hydrolysates from cutlassfish heads can serve as peptones for microbial culture media, highlighting their suitability as a nutrient source [27].

Therefore, this study aims to explore the additional bioactivities of cutlassfish head peptone (CP), focusing on its anti-inflammatory and antioxidant effects in lipopolysaccharide (LPS)-stimulated RAW264.7 macrophages, as well as elucidating the underlying molecular mechanisms contributing to their effects. By expanding the application scope for these peptones, this study underscores the broader value of fishery by-products in the development of functional materials and addressing chronic inflammatory conditions.

## 2. Materials and Methods

### 2.1. Preparation of Peptone from the Cutlassfish Head

Peptone was prepared using a previously described enzymatic hydrolysis method. Cutlassfish heads were provided by Hallim-Suhyup, Jeju, South Korea [27]. The heads were washed thoroughly and heated at 100 °C for 15 min. Subsequently, the muscle tissue was separated, freeze-dried, and pulverized into powder form. Hydrolysis was conducted in a shaking incubator at 180 rpm, 55 °C for 24 h by adding 5% substrate and 0.5% enzyme Protamex (Sigma Aldrich, St. Louis, MO, USA). The enzymatic reaction was inactivated by incubating the mixture at 95 °C for 10 min. The supernatant was collected through centrifugation and subsequently freeze-dried for further analysis. CP samples were prepared by dissolving 10 mg of CP in 1 mL of distilled water, filtering through a 0.45 µm filter, and subsequently diluting to the designated concentrations before use.

### 2.2. ABTS Radical Scavenging Assay

The ABTS radical scavenging assay was conducted as described previously [28]. The ABTS cation radical was generated by reacting 7.0 mM ABTS (Sigma Aldrich, USA) with a 4.95 mM potassium persulfate (Sigma Aldrich, USA) solution in the dark for 12 h at 25 °C. Before the assay, the solution was diluted with methanol until it achieved an absorbance of 1.100 ± 0.020 at 734 nm. For the assay, 100 μL of each sample was combined with 100 μL of the ABTS radical solution in 96-well plates. After 10 min incubation in the dark at room temperature, the absorbance was measured at 734 nm.

### 2.3. DPPH Radical Scavenging Activity

The DPPH scavenging assay was evaluated according to the method of Alzagameem with slight modification [29]. In the DPPH assay, 1 mL of the CP sample at the designated concentrations (2, 4, 6, 8, and 10 mg/mL) was mixed with 1 mL of 0.2 mM DPPH (Millipore, USA) in ethanol. The mixture was incubated in the dark at 25 °C for 30 min. The absorbance of the mixed solution was then measured at 517 nm using a spectrophotometer.

### 2.4. Ferric-Reducing Antioxidant Power Assay (FRAP)

The FRAP assay was performed using the protocol described by earlier researchers [30]. FRAP reagent was prepared each time by combining 0.3 M acetate buffer (pH 3.6) and 0.04 M TPTZ (Sigma Aldrich, USA) in 40 mM HCl, 0.02 M FeCl_3_ (Sigma Aldrich, USA) in the ratio of 10:1:1. The concentration of CP in 200 μL was prepared at 2, 4, 6, 8, and 10 mg/mL. Subsequently, 200 μL of CP and 150 μL of freshly prepared FRAP reagent were mixed in a 96-well plate and incubated for 10 min at 37 °C. The absorbance was read at 593 nm.

### 2.5. Cell Culture and Cell Viability Assay

RAW 264.7 macrophages (passages 5–10) were obtained from the Korean Cell Line Bank (KCLB, Republic of Korea) and cultured in Dulbecco’s modified Eagle’s medium (DMEM; Gibco, New York, NY, USA) supplemented with 10% fetal bovine serum (FBS; Gibco, USA) and antibiotics (WELGENE, Gyeongsan-si, Republic of Korea) at 37 °C in a humidified incubator with 5% CO_2_. RAW 264.7 macrophages were seeded at 2.5 × 10^4^ cells/well in 96-well plates and incubated overnight. Cells were treated with CP (0, 0.125, 0.25, 0.5, 1 mg/mL) for 24 h. The concentrations of CP (ranging from 0.125 mg/mL to 1 mg/mL) were determined based on preliminary studies assessing the optimal range for evaluating antioxidant and anti-inflammatory effects in macrophage cultures. These concentrations were selected to ensure a clear dose-dependent response while avoiding cytotoxicity. Subsequently, the cells in each well were treated with 10 μL of WST-1 reagent (Biomax, Guri-si, Republic of Korea). After 1 h, the absorbance of each well was read at 450 nm using a microplate reader (Bio-Tek Synergy HT, USA). Cell viability (%) was calculated as (OD sample/OD blank) × 100.

### 2.6. NO Production

The cells were seeded in a 96-well plate at 2.5 × 10^4^ cells/well and incubated overnight. Subsequently, the cells were pre-incubated with CP (0.125, 0.25, 0.5, and 1 mg/mL) for 2 h, followed by 1 μg/mL LPS (Sigma Aldrich, USA) stimulation for 24 h. The NO content was measured in the culture supernatant using Griess reagent (Promega, USA) according to the manufacturer’s instructions.

### 2.7. Real-Time PCR

The cells were seeded at 7 × 10^5^ cells/well in a 6-well plate and incubated overnight. After treatment with CP (0.125, 0.25, 0.5, and 1 mg/mL) for 2 h and stimulation with 1 μg/mL LPS (Sigma Aldrich, USA) for 24 h, total RNA was extracted using Trizol reagent and synthesized into cDNA using the PrimeScript™ RT reagent kit with a gDNA Eraser (Takara, RR047A), following the manufacturer’s instructions. Real-time qPCR was conducted on a QuantStudio 3 Real-Time PCR System (ThermoFisher Scientific, Waltham, MA, USA) using PowerUp SYBR Green Master Mix (ThermoFisher Scientific, USA). Gene expression levels relative to GAPDH were evaluated using the ΔΔCt method. The primer sequences used for Real-Time PCR were adopted from previously published studies [31]. The primer sequences are shown in Table 1.

### 2.8. Western Blot Analysis

The cells were lysed in a radioimmunoprecipitation assay buffer with a protease inhibitor for 30 min on ice. The protein concentration was measured using the Pierce™ BCA protein assay kit (Thermo Fisher Scientific, USA). Subsequently, 20 μg of proteins were electrophoresed through 4–12% sodium dodecyl sulfate–polyacrylamide gel electrophoresis gels and transferred onto a polyvinylidene difluoride membrane. The membrane was blocked for 1 h at RT with 5% BSA in TBS-T, followed by overnight incubation at 4 °C with primary antibodies, including iNOS (Cell Signaling Technology, Inc. (Danvers, MA, USA), #2977, 1:1000), COX-2 (Cell Signaling, #4842, 1:1000), Phospho-p38 (Cell Signaling, #4511, 1:500), p38 (Cell Signaling, #8690, 1:1000), JNK (Cell Signaling, #9252, 1:1000), Phospho-JNK (Cell Signaling, #9251, 1:500), ERK (Cell Signaling, #4695, 1:1000), Phospho-ERK (Cell Signaling, #4370, 1:500), and GAPDH (Cell Signaling, #5174, 1:1000). After washing with TBS-T, the membrane was incubated for 1 h at RT with secondary antibodies, including Goat anti-rabbit IgG (ThermoFisher Scientific, #31460, 1:5000) and Goat anti-mouse IgG (ThermoFisher Scientific, #31430, 1:5000). The proteins were visualized using an Amersham Imager 600 (GE Healthcare, Chicago, IL, USA) and quantified with the ImageJ (version 1.54k) program.

### 2.9. Reactive Oxygen Species (ROS) Measurement

RAW 264.7 macrophages were seeded into 96-well plates and pretreated with or without CP at concentrations ranging from 0.125 to 1 mg/mL for 2 h, followed by adding LPS (1 μg/mL) for 24 h. Subsequently, the cells were incubated with 10 µM DCFH-DA (Sigma Aldrich, USA) for 30 min in the dark. The fluorescence intensity was measured at excitation and emission wavelengths of 485 and 535 nm, respectively, using a microplate reader.

### 2.10. Data Analysis

Statistical analysis was conducted with GraphPad Prism software version 10.3.1 (San Diego, CA, USA). A one-way analysis of variance was used to compare means, and the results were expressed as the mean ± standard deviation. Differences between means were separated using Duncan’s multiple range test at *p* < 0.05.

## 3. Results

### 3.1. In Vitro Antioxidant Activity of CP

**Figure 1:**
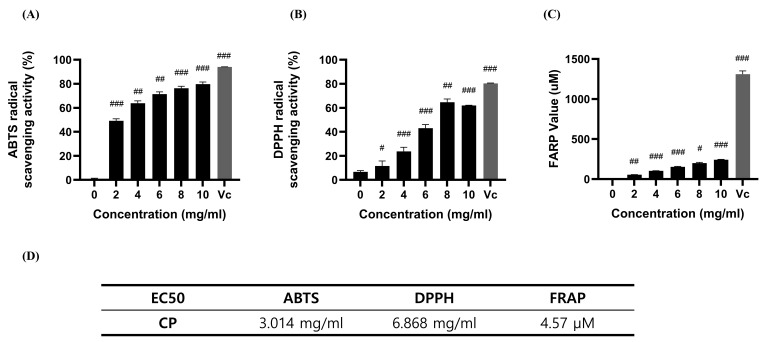
Antioxidant activities of CP and vitamin C (Vit. C, 0.1 mg/mL). (**A**) ABTS radical scavenging activity. (**B**) DPPH radical scavenging activity. (**C**) FRAPP assay. Vit. C (0.1 mg/mL) was used as the control. Vc: vitamin C. Data are shown as the mean ± standard deviation (SD) (*n* = 3). ^#^ *p* < 0.05, ^##^ *p* < 0.01, and ^###^ *p* < 0.001 versus the control group. (**D**) EC_50_ values for ABTS, DPPH, and FRAP assays.

The results of the antioxidant activity of CP are presented in the ABTS radical scavenging activity of CP, demonstrating inhibition in the range of 79.66% to 49.14% at concentrations of 10, 8, 6, 4, and 2 mg/mL, with a half-maximal effective concentration (EC50) value of 1.855 mg/mL (Figure 1A). The maximum DPPH radical scavenging activity of CP at a concentration of 8 mg/mL was 64.69%. However, at higher concentrations, the activity decreased (Figure 1B). According to the FRAP assay, CP exhibited a maximum potential of 224.54 μM for the reduction of ferric ions to ferrous ions at a concentration of 10 mg/mL.

### 3.2. Effect of CP on Cell Viability

**Figure 2:**
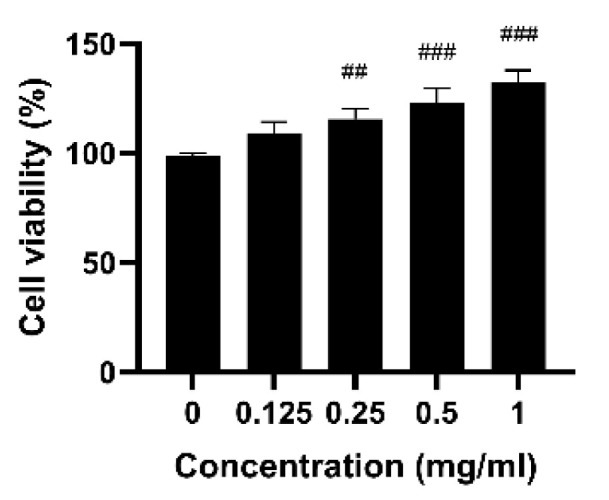
Effects of CP on proliferation in RAW 264.7 cells. The results are presented as the mean ± SD from three independent experiments. ^##^ *p* < 0.01 and ^###^ *p* < 0.001 versus the control group.

The cytotoxicity of CP in RAW 264.7 cells was evaluated using the WST assay. Cell viability was determined after treating the RAW 264.7 cells with CP (0.125, 0.25, 0.5, and 1 mg/mL). CP induced no cytotoxicity and significantly enhanced cell proliferation in a dose-dependent manner (Figure 2). Under treatment with 1 mg/mL CP, cell growth was increased by more than 33.3% compared with that under the control treatment.

### 3.3. CP Inhibited NO Production in LPS-Treated RAW264.7 Macrophages

**Figure 3:**
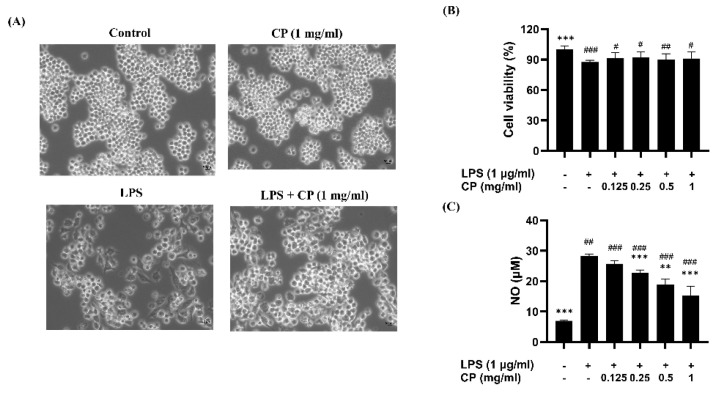
Effects of CP on lipopolysaccharide (LPS)-induced nitric oxide (NO) production. (**A**) The cell morphology was visualized under an inverted-phase contrast microscope (×200). (**B**) Cell viability was evaluated after the cells were pretreated with varying CP concentrations for 2 h, followed by LPS stimulation (1 μg/mL) for 24 h. (**C**) NO production was determined using Griess reagent. The data are presented as the mean ± SD. ^#^ *p* < 0.05, ^##^ *p* < 0.01, and ^###^ *p* < 0.001 versus the control group. ** *p* < 0.01 and *** *p* < 0.001 versus the LPS-treated group.

To determine the effect of CP on NO production during LPS-induced inflammation, the cells were pretreated with CP for 2 h and stimulated with LPS for 24 h. Upon LPS stimulation, the RAW 264.7 macrophages exhibited activation characterized by increased cell size, morphological changes to a spindle or dendritic shape, enhanced adhesion, and a more spread-out morphology, which were abrogated by CP treatment (Figure 3A). LPS stimulation markedly elevated NO production. In contrast, pretreatment with CP significantly attenuated this increase in a dose-dependent manner (Figure 3C), while there was no significant difference in cell viability (Figure 3B).

### 3.4. CP Inhibited ROS Production in LPS-Treated RAW 264.7 Macrophage Cells

**Figure 4:**
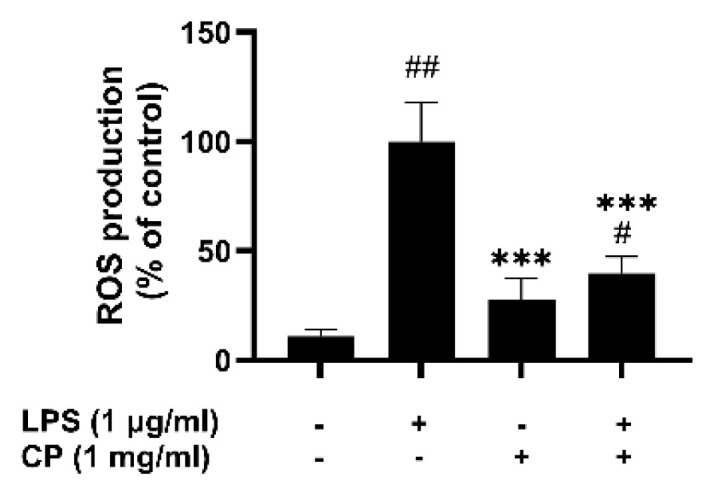
Effects of CP on LPS-induced reactive oxygen species (ROS) production. RAW 264.7 cells were treated with 1 μg/mL LPS for 24 h in the presence or absence of CP pretreatment for 2 h and stained with DCFH-DA for 30 min at 37 °C to induce ROS production. The ROS production levels were determined using a fluorescence microplate reader. Data are shown as the mean ± SD of three replicates. ^#^ *p* < 0.05 and ^##^ *p* < 0.01 versus the control group, *** *p* < 0.001 versus the LPS-treated group.

Considering the involvement of ROS in driving the production of inflammatory mediators during the inflammatory response, the effect of CP on ROS generation was evaluated using a fluorescence microplate reader. ROS levels were significantly increased in the RAW 264.7 cells treated with LPS compared with those in the blank control group (Figure 4). However, pretreatment with CP (1 mg/mL) reduced the ROS levels by 60% compared with those in the LPS-treated group, suggesting that CP inhibits ROS production during the inflammatory response.

### 3.5. CP Inhibited the Expression of COX-2 and Inducible Nitric Oxide Synthase (iNOS) in the LPS-Treated RAW 264.7 Macrophage Cells

**Figure 5:**
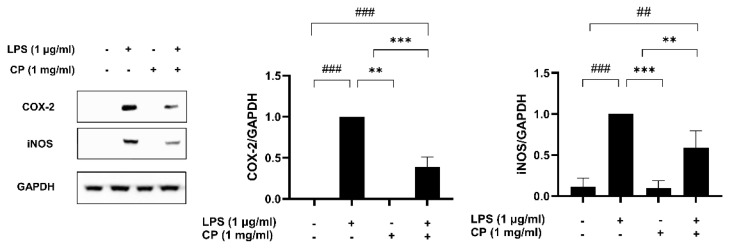
Effects of CP on inducible nitric oxide synthase (iNOS) and cyclooxygenase-2 (COX-2) expression in RAW 264.7 cells. The effects of CP on the expression of iNOS and COX-2 proteins induced by LPS were examined. Cells were pretreated with 1 mg/mL CP for 2 h and stimulated with 1 μg/mL LPS. After 24 h of incubation, the iNOS and COX-2 protein levels were assessed via Western blotting. Western blot bands were quantified using ImageJ and normalized to the loading control. Values are presented as the mean ± SD of three independent experiments. ^##^ *p* < 0.01 and ^###^ *p* < 0.001 versus the control group. ** *p* < 0.01 and *** *p* < 0.001 versus the LPS-treated group.

CP inhibited the expression of COX-2 and iNOS in the LPS-treated RAW264.7 macrophage cells. COX-2 and iNOS expression levels were investigated in the LPS-induced RAW 264.7 macrophages to assess the anti-inflammatory effect of CP. The protein levels of iNOS and COX-2 were undetectable in the untreated RAW 264.7 cells (Figure 5). However, in the group treated with LPS alone, the protein levels of iNOS and COX-2 were significantly increased. Notably, pretreatment with CP significantly suppressed the LPS-induced expression of iNOS and COX-2 proteins.

### 3.6. CP Inhibited the mRNA Levels of the Pro-Inflammatory Cytokines IL-1β and IL-6

**Figure 6:**
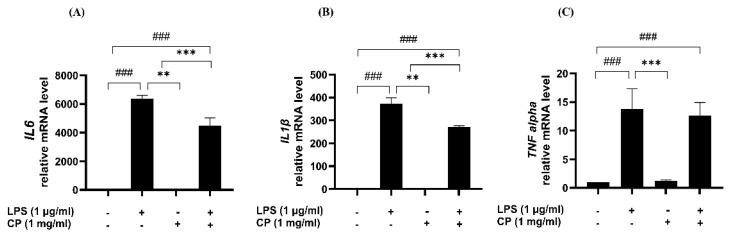
Inhibitory effects of CP on LPS-stimulated pro-inflammatory cytokine expression in RAW 264.7 cells. The cells were treated with CP (1 mg/mL) for 2 h and LPS (1 µg/mL) for 24 h. The pro-inflammatory cytokine mRNA levels were measured using RT-qPCR. (**A**) IL-6, (**B**) IL-1β, and (**C**) TNF-α levels. Values are the means ± SD of three independent experiments. ^###^ *p* < 0.001 versus the control group, ** *p* < 0.01, and *** *p* < 0.001 versus the LPS-treated group.

Subsequently, RT-qPCR was used to evaluate the effects of CP on LPS-induced inflammatory responses. The mRNA expression levels of IL-6, IL-1β, and TNF-α were significantly upregulated in the LPS-treated cells compared with those in the control group (Figure 6). However, in the CP-pretreated group, the expression levels of IL-6 and IL-1β were significantly suppressed, whereas those of TNF-α remained unaffected by CP pretreatment.

### 3.7. Effects of CP on the Activation of Mitogen-Activated Protein Kinases (Mapks) in LPS-Stimulated RAW 264.7 Cells

**Figure 7:**
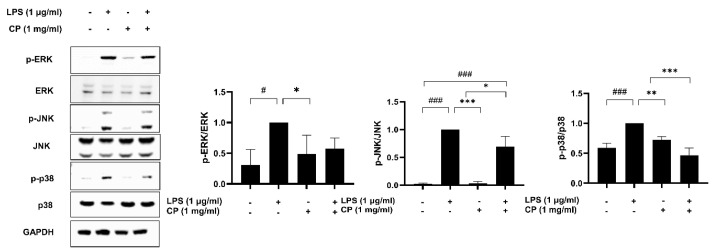
Effects of CP on MAPK activation in RAW 264.7 cells. The RAW 264.7 cells were pretreated with 1 mg/mL CP for 2 h, followed by stimulation with 1 μg/mL LPS for 24 h. The protein expression levels of total and phosphorylated ERK, JNK, and p38 in the LPS-induced RAW 264.7 cells were determined using Western blotting, and GAPDH was used as a loading control. Densitometric analysis of phosphorylated protein levels was performed using ImageJ software and normalized to total protein levels. Values are the means ± SD of three independent experiments. ^#^ *p* < 0.05 and ^###^ *p* < 0.001 versus the control group. * *p* < 0.05, ** *p* < 0.01, and *** *p* < 0.001 versus the LPS-treated group.

MAPKs are critical regulators in immune cell activation, inflammatory cytokine production, and the overall inflammatory response through the modulation of cellular signaling pathways [32,33]. These biological processes are activated by LPS stimulation [34]. Therefore, we investigated the effect of CP on the LPS-induced phosphorylation of MAPKs in RAW 264.7 macrophages using Western blotting. The results showed that compared with those of the LPS-stimulated group, the LP-induced phosphorylation levels of p38, JNK, and ERK were suppressed in the CP pretreatment group (Figure 7). These findings suggest that the inhibition of p38, JNK, and ERK phosphorylation may be involved in the suppression of LPS-induced inflammatory responses in RAW 264.7 macrophages.

## 4. Discussion

This study demonstrates that CP exhibits strong antioxidant and anti-inflammatory effects in LPS-stimulated RAW 264.7 macrophages. These findings support the bioactive potential of fishery by-products in modulating inflammatory responses and oxidative stress.

In antioxidant assays, CP exhibited antioxidant activity comparable to that of Vitamin C, particularly in the ABTS assay, where it demonstrated a strong ability to scavenge free radicals (Figure 1). This highlights CP as a potential natural alternative for managing oxidative stress, a key factor in the development of chronic diseases. Since the ABTS assay evaluates both water- and lipid-soluble antioxidants, whereas the DPPH assay primarily assesses lipid-soluble antioxidants [35], these results suggest that CP may exhibit higher antioxidant activity in hydrophilic environments. Given that protein hydrolysates often contain bioactive peptides with antioxidant properties [36], peptides in CP may contribute to this activity, particularly through water-soluble peptides.

Additionally, CP promoted RAW 264.7 macrophage proliferation, possibly due to the bioactive peptides and amino acids in the peptone. These components are known to influence cell proliferation and metabolism and have been reported to enhance cellular activity [37,38]. Consistently, previous studies have shown that fish peptone promotes Caco-2 cell proliferation in a concentration-dependent manner, further supporting the findings of this study [39].

CP significantly inhibited the production of NO and ROS in LPS-stimulated RAW 264.7 macrophages, indicating its potential to modulate oxidative stress and inflammation [40]. Moreover, CP suppressed the expression of COX-2 and iNOS, enzymes responsible for producing pro -inflammatory mediators such as prostaglandins and NO [41]. The significant downregulation of these enzymes suggests that CP may modulate inflammatory pathways at the molecular level. These findings are consistent with those of earlier research on fish-derived peptides showing similar reductions in iNOS and COX-2 expression [42].

The reduction in ROS and NO production, as well as the suppression of COX-2 and iNOS expression, suggests that CP could offer a potential therapeutic strategy for managing chronic inflammatory diseases like rheumatoid arthritis and cardiovascular conditions. This action could be harnessed in the development of functional food products or pharmaceutical supplements aimed at reducing inflammation and improving overall health.

The inhibition of MAPK phosphorylation by CP highlights a key mechanism underlying its anti-inflammatory effects. The MAPK signaling pathway, particularly ERK, p38, and JNK phosphorylation, plays a central role in regulating inflammatory responses and cytokine production [34]. CP’s suppression of MAPK phosphorylation suggests an upstream role in the inflammatory cascade, possibly through inhibition of the TLR4/NF-κB pathway and subsequent blockade of transcription factors regulating pro-inflammatory gene expression. This mechanism has also been observed in other marine-derived bioactive compounds [24,43]. CP’s effects on the three MAPKs provide further insights into its regulation of inflammatory signaling.

Furthermore, CP selectively inhibited the expression of IL-1β and IL-6, which are crucial mediators in chronic inflammation [16,24], without affecting TNF-α. The lack of TNF-α inhibition suggests that CP may selectively modulate cytokine pathways downstream of MAPK signaling rather than broadly suppressing all inflammatory mediators. This selectivity highlights the potential of CP in targeting specific inflammatory responses while minimizing broad-spectrum immunosuppression.

Recent studies have highlighted the therapeutic potential of bioactive peptides derived from various marine organisms, including fish, crustaceans, mollusks, algae, corals, and microorganisms [15,24,44,45,46]. These peptides exhibit diverse biological activities and are increasingly recognized for their applications in functional foods and pharmaceuticals.

For instance, a peptide derived from salmon has been reported to possess anti-inflammatory properties [22], a tetrapeptide from *Palmaria palmata* has demonstrated anti-atherosclerotic effects [47], and a peptide extracted from *Crassostrea gigas* has exhibited anticoagulant activity [48]. These findings underscore the significance of marine-derived peptides in biomedical applications.

Emerging evidence suggests that low-molecular-weight peptides (<1 kDa) enhance cellular permeability via transporter proteins (e.g., PEPT1 and PEPT2) and interact with intracellular targets [49,50], thereby potentiating bioactivity. Zou et al. previously reported that low-molecular-weight peptides exert strong antioxidant and anti-inflammatory effects by scavenging reactive oxygen species (ROS) and modulating NF-κB and MAPK signaling pathways [51].

In accordance with these findings, the biological activity of CP appears to be mediated by peptides, particularly low-molecular-weight peptides that contribute to its functional properties. This suggests that CP may share mechanisms similar to those of other marine-derived bioactive peptides.

Despite its promising bioactivity, the precise composition of CP, including specific peptide sequences and molecular characteristics, remains unclear. Further studies are required to identify the key bioactive peptides responsible for CP’s effects and to establish their structure–activity relationships. A comprehensive understanding of these properties will provide deeper insights into CP’s mechanism of action and facilitate its potential applications.

Considering CP’s strong bioactivity, its potential extends beyond biomedical applications to large-scale industrial utilization. Successful commercialization requires the optimization of large-scale production while ensuring feasibility, scalability, cost-effectiveness, sustainable raw material supply, and regulatory compliance [8,52,53]. Enzymatic hydrolysis should be optimized to maximize peptide yield while preserving bioactivity.

Although cutlassfish by-products serve as a sustainable raw material, CP’s stability during storage and processing remains a significant challenge. Protein hydrolysates are prone to oxidation and proteolysis, potentially compromising their bioactivity [54]. Further research is required to enhance CP’s bioavailability and shelf stability. Advanced processing techniques, such as freeze-drying and microencapsulation, should be investigated to mitigate degradation under various environmental conditions [55].

Future research should focus on elucidating CP’s bioactive mechanisms and optimizing its industrial feasibility. Additionally, in vivo and clinical studies are essential to validate its long-term efficacy, safety, and pharmacokinetics. Standardized production protocols and regulatory compliance will be critical for its successful commercialization in pharmaceuticals and functional foods.

## 5. Conclusions

CP, derived from cutlassfish heads, demonstrates potent antioxidant and anti-inflammatory properties, making it a promising candidate for therapeutic applications in managing oxidative stress and inflammation. Further studies on its bioavailability and efficacy in vivo are necessary to fully explore its potential in functional foods and pharmaceuticals.

## Figures and Tables

**Table 1:** Real-Time PCR primer sequences.

Genes	Number	Primer Sequences
IL-1β	NM_000576	F 5′-CAGGATGAGGACATGAGCACC-3′
		R 5′-CTCTGCAGACTCAAACTCCAC-3′
IL-6	NM_000600	F 5′-GTACTCCAGAAGACCAGAGG-3′
		R 5′-TGCTGGTGACAACCACGGCC-3′
TNF-α	NM_000594	F 5′-TTGACCTCAGCGCTGAGTTG-3′
		R 5′-CCTGTAGCCCACGTCGTAGC-3′
GAPDH	NM_002046	F 5′-AAGGGTCATCATCTCTGCCC-3′
		R 5′-GTGATGGCATGGACTGTGGT-3′

## Data Availability

Data from this study are available from the corresponding author upon reasonable request.

## Abbreviations

The following abbreviations are used in this manuscript:

CP	Cutlassfish head peptone
ROS	Reactive oxygen species
DPPH	2,2-diphenyl-1-picrylhydrazyl
FRAP	Ferric-reducing antioxidant power
COX-2	Cyclooxygenase-2
LPS	Lipopolysaccharide
